# Pre-COVID respiratory sinus arrhythmia moderates associations between COVID-19 stress and child externalizing behaviors: Testing neurobiological stress theories

**DOI:** 10.1017/S0954579423001682

**Published:** 2024-01-26

**Authors:** Hilary Skov, Erin B. Glackin, Stacy S. Drury, Jeffrey Lockman, Sarah A. O. Gray

**Affiliations:** 1 Department of Psychological Sciences, University of Connecticut, Storrs, CT, USA; 2 Department of Psychology, Tulane University School of Science and Engineering, New Orleans, LA, USA; 3 Department of Psychiatry and Behavioral Science, Boston Children’s Hospital, Boston, MA, USA; 4 Department of Psychiatry, Harvard Medical School, Boston, MA, USA

**Keywords:** COVID-19 pandemic, diathesis stress, respiratory sinus arrhythmia, stress, biological sensitivity to context

## Abstract

Exposure to stress related to the COVID-19 pandemic contributes to psychopathology risk, yet not all children are negatively impacted. The current study examined a parasympathetic biomarker of stress sensitivity, respiratory sinus arrhythmia (RSA), as a moderator of the effects of exposure to pandemic stress on child internalizing and externalizing behaviors in a sample of children experiencing economic marginalization. Three to five years pre-pandemic, when children were preschool-aged, RSA during baseline and a challenging parent-child interaction were collected. Mid-pandemic, between November 2020 and March 2021, children’s exposure to pandemic stress and internalizing and externalizing behaviors were collected. Results demonstrated that children who, pre-pandemic, demonstrated blunted parasympathetic reactivity (i.e., no change in RSA relative to baseline) during the dyadic challenge exhibited elevated risk for externalizing behaviors mid-pandemic. Further, this risk was greatest for children exposed to high and moderate levels of pandemic stress. Consistent with diathesis stress and polyvagal frameworks, these conditional effects suggest that blunted parasympathetic reactivity in response to stress in early childhood may escalate the development of externalizing behaviors following stress exposure at school age.

## Introduction

The COVID-19 pandemic and subsequent mitigation strategies have caused large-scale, multisystem disruptions, exacting a toll on youth mental health. Relative to pre-COVID norms, researchers have observed increases in depression, anxiety, inattention, and/or oppositional/defiant behaviors across samples of young children (Glynn et al., 2021), school- aged children (Rosen et al., 2021; Weissman et al., 2021), and adolescents (Breaux et al., 2021; Duan et al., 2020; Minihan et al., 2022; Rosen et al., 2021). Notably, the degree of pandemic-related stress exposure has varied widely, with low-income and economically marginalized (LIEM) communities bearing a disproportionate burden. Relative to their higher-income counterparts, LIEM communities have experienced higher rates of cases, hospitalizations, and death (Parenteau et al., 2022), increased barriers to physical distancing (e.g., needing to work outside the home; Jay et al., 2020), higher rates of unemployment and job insecurity (Parenteau et al., 2022), and increased housing instability (Greens & McCargo, 2020). For LIEM families living in New Orleans, pandemic-related challenges may build on repeated exposure to various stressors (e.g., community violence, natural disasters; Drury et al., 2008; Zacher et al., 2023).

Consistent with the COVID-19 family disruption model (Prime et al., 2020), stress exposure both prior to and during the pandemic correspond to the degree of children’s symptom severity (Shoychet et al., 2023). For example, in another sample of young children, those with previous exposure to trauma and current exposure to stressors had elevated externalizing behaviors relative to those previously exposed to trauma without current life stress (Grasso et al., 2013). Although cumulative stress exposure increases risk for mental health difficulties during the pandemic, it does not independently determine outcomes. Thus, the present study examines if and how physiological stress activity buffers against or exacerbates risk for mental health difficulties following exposure to pandemic-related stress in a sample of LIEM children in New Orleans.

## The moderating role of physiological stress sensitivity

It is well-established that the activity of the autonomic nervous system (ANS), which modulates physiological arousal, contributes to variability in children’s post-exposure outcomes (Cipriano-Essel et al., 2013; Eisenberg et al., 2012; Skowron et al., 2014; Gray et al., 2017). The two branches of the ANS are the parasympathetic (i.e., “rest and restore” system; PNS) and sympathetic nervous system (i.e., “fight or flight” system; SNS), which work in tandem to maintain heartbeat, respiration, digestion, and other automatic bodily functions (Beauchaine, 2015). Respiratory sinus arrhythmia (RSA) is a measure of PNS activity capturing the variability in heart rate across the respiration cycle. PNS activity, measured via RSA, is implicated in stress- related outcomes due to its role in downregulating states of high physiological arousal, such as fear and anger.

Baseline levels of RSA, measured at rest, are thought to capture an individual’s “capacity for regulation” by measuring tonic PNS control over heart rate (Beauchaine, 2015). Consistent with this idea, average levels of baseline RSA change across development, but rank order of individual differences in baseline levels of RSA are relatively stable from toddlerhood through adolescence (Dollar et al., 2020). According to polyvagal theory, higher levels promote rest and perception of the environment as safe, while lower levels may represent dysregulation or disengagement (Porges, 2007). Indeed, meta-analytic research from infancy through adolescence links lower levels of baseline RSA with higher levels of mental health symptoms across diagnostic criteria (Graziano & Derefinko, 2013). Similarly, in another sample of young children exposed to Hurricane Katrina, lower levels of pre-disaster RSA prospectively predicted higher levels of posttraumatic stress disorder (PTSD) symptoms (Mikolajewski & Scheeringa, 2018). Thus, low levels of baseline RSA, or PNS influence on an individual’s heart rate under neutral conditions, may enhance risk for mental health difficulties.

Patterns of RSA reactivity (i.e., task-based change in RSA, relative to baseline) provide insight into a child’s response to challenging or stressful situations through capturing fluctuations in PNS activity (Beauchaine, 2015). According to polyvagal theory, RSA withdrawal (i.e., a decrease in RSA relative to baseline) in response to challenge or stress promotes attentional and behavioral control, while RSA augmentation (i.e., an increase in RSA activity relative to baseline) or blunted RSA (i.e., no change in RSA activity relative to baseline) represents a failure to engage with or respond to environmental stress (Porges, 2007). In alignment with this framework, meta-analytic research has found that greater levels of RSA withdrawal are related to fewer internalizing and externalizing behaviors across samples of participants spanning infancy through adolescence (Graziano & Derefinko, 2013). Across child development, research largely supports the idea that withdrawal of PNS influence represents an efficient and adaptive response to environmental demands through suppressing restorative processes without recruiting SNS resources.

Developmental theories posit that patterns of physiological stress sensitivity prior to stress exposure increase vulnerability to mental health difficulties following that exposure. The present investigation is informed by two distinct yet overlapping neurobiological models positing that patterns of RSA activity may underlie sensitivity to COVID stress: the diathesis stress (DS) and biological sensitivity to context (BSC) theories. DS theory posits that patterns of physiological sensitivity prior to stress exposure increase vulnerability to, or the likelihood of, mental health difficulties following that exposure (Belsky & Pluess, 2009). BSC expands upon DS through positing that individuals who are more sensitive to stress exposure may also be disproportionately susceptible to the benefits of supportive environments (including environments with relative absence of risk; Belsky & Pluess, 2009). Specifically, BSC supports the idea that stress response system activity can either enhance or buffer the effect of exposures in a “for better or worse” manner (Boyce & Ellis, 2005). Guided by these theories, the present study prospectively tests the moderating role of pre-pandemic physiological sensitivity (i.e., baseline RSA and RSA reactivity) on associations between COVID stress exposure and child externalizing and internalizing behaviors.

## Distinguishing theories of neurobiological stress sensitivity

In early and middle childhood, there are mixed findings regarding if and how stress exposure and physiological sensitivity interact to predict mental health symptoms. Certain cross-sectional findings have demonstrated partially attenuated interactions consistent with polyvagal theory. In these interactions, simple slopes of associations between stress exposure and mental health symptoms would be positive both for children with high and low physiological sensitivity, but one slope would be steeper (Sommet et al., 2023). For example, lower baseline RSA significantly increased the strength of positive associations among childhood violence and externalizing behaviors for boys only (El-Sheikh & Hinnant., 2011). Similarly, in a sample of preschoolers, domestic violence exposure and externalizing behaviors were positively associated, and this association was stronger for children who exhibited RSA augmentation during a dyadic task (Katz, 2007). Other studies examining externalizing behaviors as an outcome found fully attenuated (i.e., ordinal) interactions that supported both diathesis stress and polyvagal theories, such that children with low physiological sensitivity faced elevated risk for difficulties following stress exposure. For example, in a cross-sectional study of preschool-aged children, cumulative stress exposure was associated with an increased risk for externalizing behaviors, but only for those with blunted or augmented RSA reactivity; no effects of cumulative stress, RSA reactivity, or their interaction were observed on internalizing behaviors (Salisbury et al., 2020). Similarly, degree of familial risk corresponded with the degree of school-age children’s externalizing problems for those with low, but not high RSA withdrawal (El-Sheikh et al., 2001). Taken together, results suggest that lower physiological sensitivity (i.e., relatively low levels of baseline RSA and/or relatively low levels of RSA withdrawal, blunted RSA, or RSA augmentation) enhance risk for externalizing behaviors in contexts of stress.

Regarding internalizing behaviors, attenuation effects are either not observed or observed in the opposite direction, such that high physiological sensitivity delineates risk. For example, in cross-sectional research with preschool-aged children, parenting stress was positively associated with internalizing (but not externalizing) behaviors, and this association was strongest for children with high levels of baseline RSA (Davis et al., 2017). Further, longitudinal work considering these associations in the context of maternal psychopathology found that RSA withdrawal was conditionally and positively associated with internalizing behaviors in middle childhood for girls whose mothers reported high levels of internalizing behaviors (Shanahan et al., 2014). However, in contexts of low stress exposure, RSA withdrawal may be associated with *less* internalizing behaviors. In preschool children who were exposed to low levels of family violence, RSA withdrawal was associated with lower levels of emotional problems; children exposed to high levels of family violence had high levels of emotional problems, regardless of RSA activity (Cipriano-Essel et al., 2013). In sum, these findings suggest that RSA withdrawal may represent adaptive and flexible engagement in contexts of low stress, whereas in contexts of high stress, it may indicate hypervigilance, dysregulated attention, or emotional lability.

Recent transdiagnostic evidence supports BSC’s assertion that physiological sensitivity can either enhance or buffer the effect of exposures in a “for better or worse” manner. In studies that found these crossover interactions, simple slopes of associations between stress exposure and mental health symptoms went in opposite directions based on level of physiological sensitivity. For example, RSA withdrawal during a challenging dyadic interaction was associated with lower inhibitory control in a sample of young children exposed to maltreatment, while RSA withdrawal was associated with higher inhibitory control for children who were not exposed to maltreatment (Skowron et al., 2014). Additionally, in 5- and 6-year-olds, greater RSA withdrawal during a dyadic challenge was associated with more externalizing behaviors in the context of high family adversity, but with less externalizing behaviors in the context of low family adversity; physiological sensitivity and family adversity did not co-contribute to internalizing behaviors (Obradović et al., 2010). However, in a sample of 5–16-year-olds, RSA withdrawal was associated with more internalizing behaviors in contexts of high adversity, and with less internalizing behaviors in contexts of low adversity for girls; no associations were present for boys (Gray et al., 2017). These crossover interactions suggest that high levels of physiological sensitivity may increase awareness to and impact of environmental context, and thus may enhance risk for mental health difficulties in high stress environments, yet buffer against them in low stress environments.

## The current study

**Figure 1.**
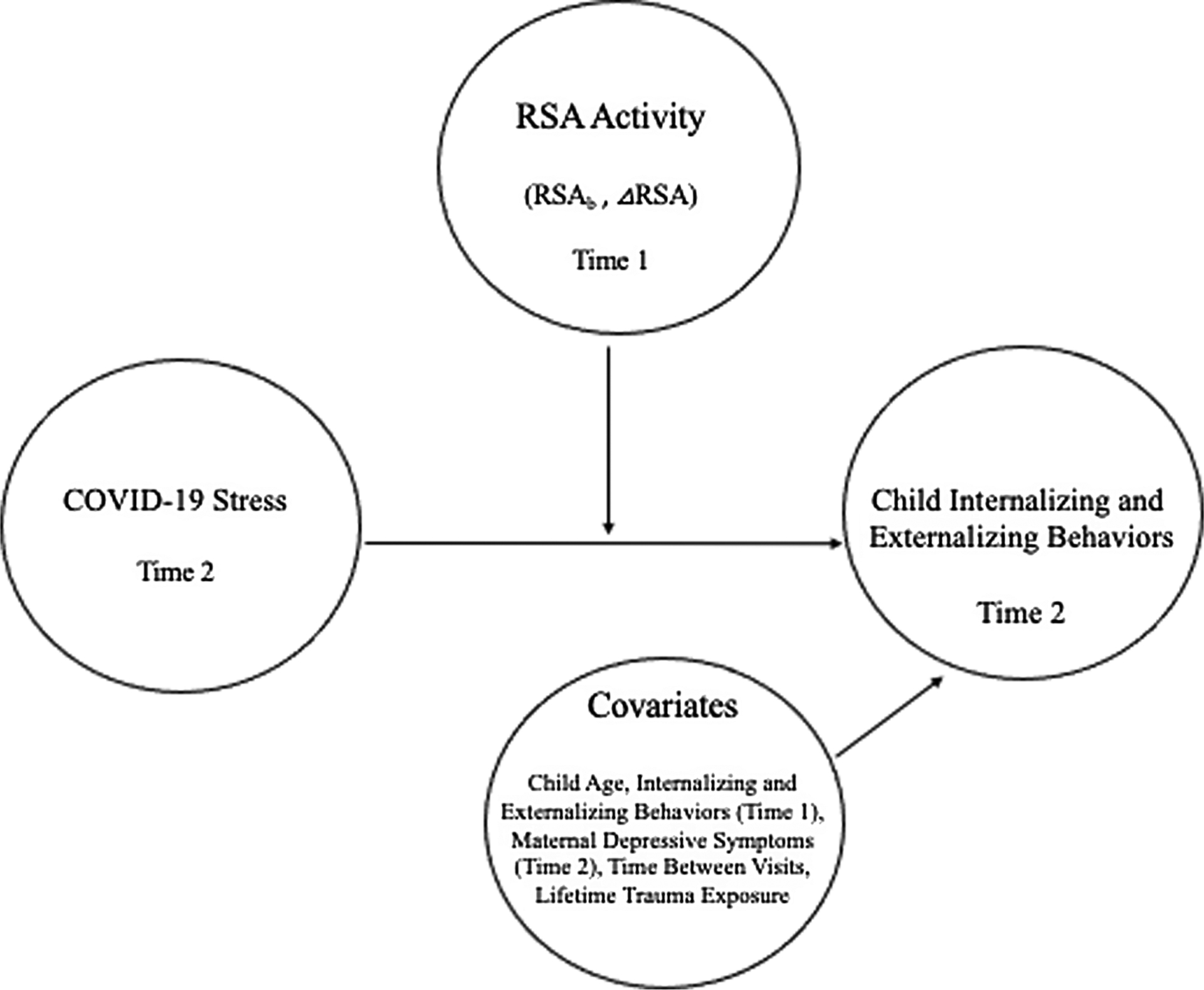
Proposed moderation model. *T1* = pre-pandemic (Time 1); *T2* = mid-pandemic (Time 2); *RSA*_*b*_ = baseline RSA; *RSAΔ* = difference in RSA from baseline to task.

The current study used a multimodal, longitudinal design to investigate *if* and *how* physiological sensitivity (i.e., baseline RSA and RSA reactivity) obtained in early childhood moderated the effect of COVID stress exposure on internalizing and externalizing behaviors at school age (See Fig. 1). In alignment with the COVID-19 family disruption model, we hypothesized that stress exposure both related and unrelated to the pandemic would be positively associated with both internalizing and externalizing behaviors. Based on the literature and several neurobiological frameworks, we also hypothesized that exposure to COVID stress would be more consequential for some children than others, and that physiological sensitivity would contribute to differences in these associations. Hypotheses regarding the strength and direction of physiological sensitivity as a moderator of these associations varied based on mental health outcome of interest.

Regarding externalizing behaviors, our hypotheses were consistent with polyvagal and diathesis-stress frameworks. In alignment with polyvagal frameworks, we hypothesized that the positive association between COVID stress exposure and externalizing behaviors in middle childhood would be strongest for children who demonstrated low levels of physiological sensitivity (i.e., relatively low levels of baseline RSA and/or relatively low levels of RSA withdrawal, blunted RSA, or RSA augmentation) in response to a dyadic stressor in early childhood. Consistent with the idea that low physiological sensitivity heightens vulnerability to externalizing behaviors under stress, we hypothesized that children with low physiological sensitivity would experience higher levels of externalizing behaviors in contexts of high, but not low, stress exposure. This would be represented through a fully attenuated (i.e., ordinal) interaction, such that the simple slope for associations between COVID stress and externalizing behaviors would be significant and positive for children with low physiological sensitivity, and simple slopes for children with high physiological sensitivity would be null (Roisman et al., 2012).

Regarding internalizing behaviors, our hypotheses were consistent with BSC frameworks. We hypothesized that the associations between COVID stress exposure and internalizing behaviors would be strongest for children who demonstrated high levels of physiological sensitivity (i.e., relatively high levels of baseline RSA and/or relatively high levels of RSA withdrawal). Consistent with the idea that high physiological sensitivity moderates associations between stress exposure and mental health outcomes in a “for better or for worse” manner, we hypothesized that the directions of these associations would vary. Specifically, children with high physiological sensitivity would experience high levels of internalizing behaviors in contexts of high stress exposure, and low levels of internalizing behaviors in contexts of low stress exposure. This would be represented through a reversed (i.e., crossover) interaction, such that the simple slope for associations between physiological sensitivity and internalizing behaviors would go in opposite directions based on level of stress exposure (Roisman et al., 2012).

## Methods

### Procedures

Data come from a larger study focused on the impacts of early life stress on young children’s social-emotional outcomes (Hatch et al., 2020). Mothers and their preschool-aged children (*N* = 175 dyads pre-pandemic at T1; September 2015–December 2018) were recruited in the New Orleans, Louisiana area from Head Start preschools, Women, Infants, and Children clinics, pediatric clinics accepting Medicaid, and similar service agencies for families experiencing economic marginalization. Mothers over the age of 18 with children between the ages of 3–5 years were eligible to participate, and those interested provided family sociodemographic information and exposure to stressors. Inclusionary criteria included that the caregiver was the child’s biological mother, and that family income was at or near the poverty line (<185%) assessed via eligibility for services. Exclusionary criteria included mothers unable to complete study measures in English and children with global developmental delay, determined by parent report of diagnosis given by a medical professional. Families were intentionally oversampled for violence exposure; all mothers who reported that they or their child had experienced or witnessed interpersonal violence were invited to participate, along with a subsample of families who reported no interpersonal violence.

During the pre-pandemic visit (T1), interested and eligible mothers reported on their own and their children’s mental health difficulties and mother and child physiological data (i.e., ECG) at rest (i.e., baseline) and during a mild, socially stressful dyadic challenge were recorded either in-home or on-site, based on mothers’ preference. During baseline, dyads were instructed to sit quietly for two minutes. During the dyadic challenge, mothers and children were presented with disassembled Duplo blocks and a picture of a complex, abstract structure that children were instructed to build in five minutes using the blocks. While mothers could provide verbal assistance, they could not provide hands-on, physical assistance. Previous studies in samples of preschool children have validated this physiological data collection paradigm (Obradović et al., 2010, 2011; Skowron et al., 2014). During the mid-pandemic remote visit (T2; November 2020–March 2021), interested and eligible mothers were consented and completed a remote survey either over the phone with a research assistant or independently online. In this survey, mothers reported on their depressive symptoms and their child’s exposure to COVID-19 stressors, cumulative trauma exposure, and mental health symptoms. Mothers received $5 for eligibility screening and $50 for each visit, while their children received a small toy for each in-person visit; a university Institutional Review Board reviewed all procedures.

### Participants

**Figure 2.**
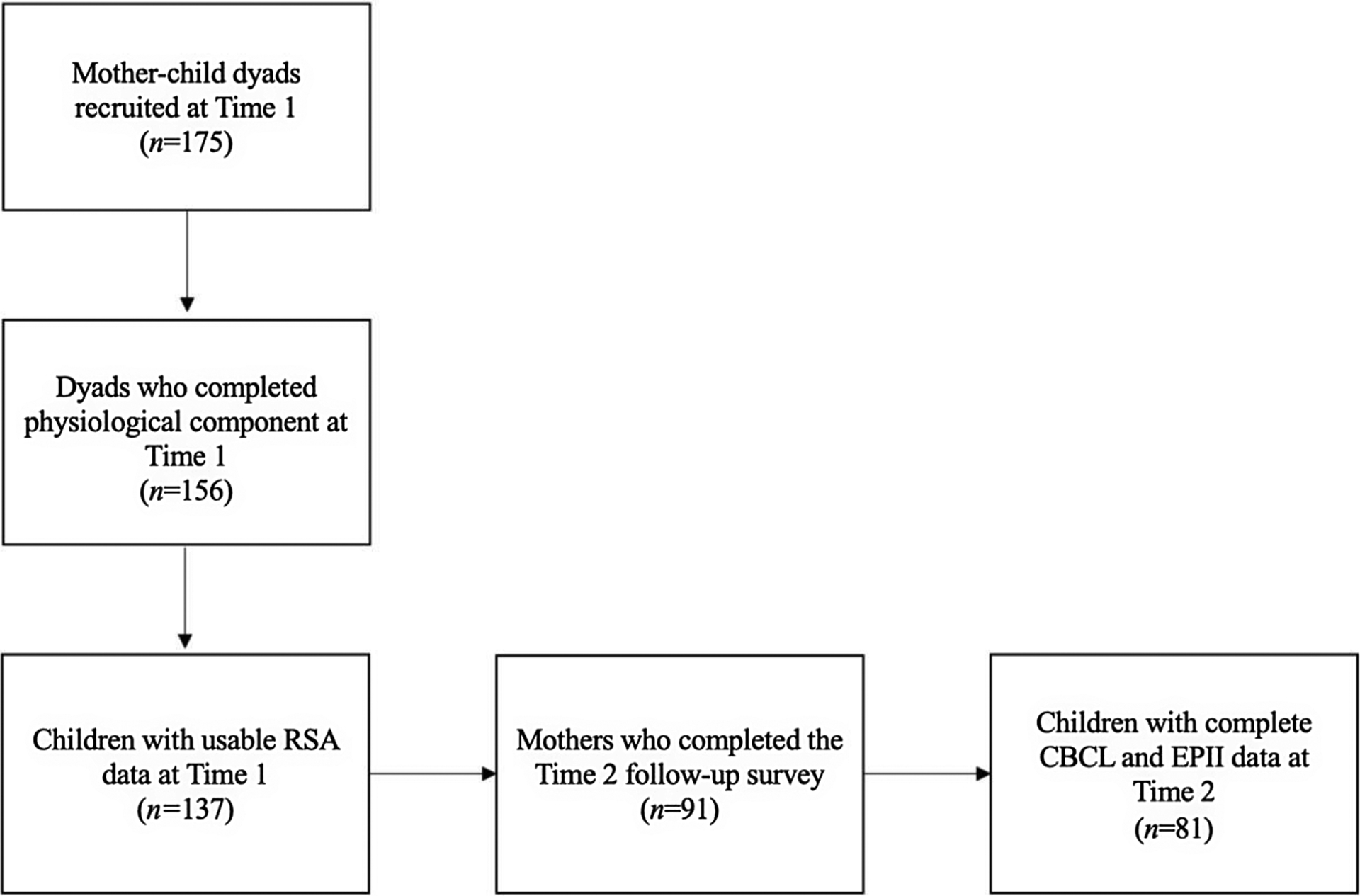
Consort diagram. *time 1* = pre-pandemic, *time* 2 = mid-pandemic; *RSA* = respiratory sinus arrythmia; *CBCL* = child behavior checklist; *EPII* = epidemic pandemic impact inventory.

Of these 175 dyads, 156 completed a second T1 visit where physiology data were collected, and 137 of these children had sufficient physiology data. Only mothers of the subsample with sufficient physiology data were recontacted for mid-pandemic follow-up (T2; *M years between visits* = 3.34, *S*.D. = .92; *Range* = *2.06*–*5.18*). Of the 137 dyads, 91 were successfully contacted and 81 had complete CBCL and COVID exposure data at T2 and were included in these analyses (see Fig. 2).

### Measures

#### Sociodemographic information

Pre-pandemic, mothers reported their own and their child’s age, race, ethnicity, and sex in a screener survey.

#### Internalizing and externalizing behaviors

Pre-pandemic, mothers completed the widely used Child Behavior Checklist (CBCL) for ages 1.5–5 (Achenbach & Rescorla, 2001) to measure their child’s externalizing and internalizing behaviors on a 3-point scale ranging from *not true (0) to very true or often true (2)*. Mid-pandemic, mothers completed the CBCL for ages 6–18 (Achenbach & Rescorla, 2000). This report used *T* scores for internalizing and externalizing behaviors (α = .86, .87; Achenbach et al., 2001). A *T* score of 60 or higher indicates that the child”s behaviors are clinically elevated or at risk of being clinically elevated.

#### Respiratory sinus arrhythmia

Pre-pandemic, ANS data were collected, filtered, extracted, and scored using Mindware software and ambulatory monitors, which the child wore in a small fannypack. Electrodes were placed on the child’s torso in a modified Lead II configuration, and leads were connected to a MindWare Technologies mobile recorder (MindWare Technologies, LTD, Westerville, OH). RSA was derived via spectral analysis of the interbeat interval series, which were detrended, tapered with a Hamming window, and subjected to Fast Fourier Transform using MindWare HRV software (version 3.1). RSA was computed as the natural log of high-frequency heart rate variability within the bandwidth associated with respiration in children (i.e., .24–1.04 HZ; Fracasso et al., 1994). Data were processed in 30-second epochs, which were visually inspected and corrected for artifacts and mismarked R peaks; 30-second epoch values were averaged across the two minutes of the baseline and five minutes of the task. No more than 10% of data was edited manually for any epoch. RSA withdrawal (ΔRSA) was calculated through a change score; the mean of children’s RSA during the dyadic task was subtracted from the mean of children’s RSA at baseline (ΔRSA = RSATask - RSABaseline). Thus, negative change scores represent children who exhibited RSA withdrawal, positive change scores represent those who exhibited RSA augmentation, and scores that do not change represent children who exhibited blunted RSA activity.

#### COVID stress exposure

Mid-pandemic, mothers reported their child’s exposure to COVID- 19-related stress using the Epidemic-Pandemic Impacts Inventory (EPII; Grasso et al., 2020). In the present study, the 32-item inventory of pandemic-related experiences specific to their child was used, which spans across several life domains including Childcare (e.g., “School or childcare was closed, or child was unable to attend”), Home life (e.g., “Exposed to more verbal and/or physical conflict between adults in the home”), Social activities and isolation (e.g., “Unable to spend time with friends in person”), and Emotional/Physical health (e.g., “Unable to access mental health treatment, medical care, and/or other supportive professional services”).

Each item has a response set of “*yes,*” “*no,*” and “*not applicable*.” Consistent with Grasso et al. (2021), three items with base rates ≤ 5% were trimmed (experienced death of a parent due to COVID, got medical treatment or was hospitalized due to symptoms of the disease), also due to concerns about measurement and double-counting: these items are captured in the cumulative trauma exposure on the Life Events Checklist described subsequently (death of a caregiver, serious illness or hospitalization). Additionally, three relatively high base-rate items (>80%) were also trimmed (i.e., school or childcare was closed or child was unable to attend, family celebrations canceled or restricted, spent more time on screens and devices) because they were pervasively experienced across the sample. For this paper, sum scores were created for COVID stressor exposure (*M* = 11.03, *SD* = 4.89, *range* = 0–24; α = .84).

#### Cumulative trauma exposure

Mothers reported on their children’s exposure to potentially traumatic events (PTEs) at mid-pandemic via a modified version of the Life Events Checklist (LEC; Gray et al., 2004), a 20-item measure capturing both direct and indirect lifetime PTE exposure (e.g., natural disaster, community violence). Each item has a response of “*yes*” or “*no*,” and sum scores were created for PTE exposure (*M* = 2.50, *SD* = 3.16, α = .88).

#### Maternal depressive symptoms

Mothers self-reported on their mid-pandemic depressive symptoms using the Center for Epidemiologic Studies Depression Scale – Revised (CESD-R; Eaton et al., 2004), a 20-item scale assessing depressed mood, disturbances with appetite or sleep, difficulty concentrating, fatigue, feelings of worthlessness, psycho-motor agitation, and suicidal ideation in the past week using a 4-point Likert scale. Sum scores were created for depressive symptoms, which fell in the clinically significant range for this sample (*M* = 18; *SD* = 18; α = .93).

### Statistical analyses

Analyses were performed using SPSS version 27. Group differences were examined by sex, race, and visit location (home vs. lab) to determine covariates using independent sample *t*- tests or one-way ANOVAs for continuous and categorical comparisons. Two bootstrapped linear regression analyses were conducted hierarchically to examine the co-contribution of COVID-19 stress and RSA on child internalizing and externalizing behaviors, with covariates and predictors entered in step one and two-way interaction terms in step two (RSA activity by COVID stress exposure) predicting internalizing and externalizing behaviors. According to gpower, our sample size provided sufficient power (80%) to detect effects of *f* ^2^ = .15 for all models, suggesting we were sufficiently powered to observe medium effect sizes (Erdfelder et al., 1996).

Statistically significant interactions were decomposed using simple slopes and slopes difference testing. Specifically, the association between the predictor and outcome was examined at high (one SD above the mean), average (mean), and low (one SD below the mean) levels of the moderator to determine whether findings were consistent with DS or BSC. Regions of significance were estimated using the Johnson-Neyman technique, which approximated the values of the predictor at which RSA activity exerted a moderating effect on the outcome. Upper and lower bounds of the regions of significance were examined to assess whether they supported BSC (i.e., were within two standard deviations of the mean; Roisman et al., 2012).

## Results

### Preliminary analyses

Descriptive statistics for mothers and children are provided in Table 1. Mothers predominantly self-identified as Black or African American (78.7%) and non-Hispanic or Latina (90.9%). The final analytic sample included 40 girls (49%) and 41 boys (51%), who were predominantly identified as Black or African American (79.8%) and non-Hispanic or Latino/a (84.5%) by their mothers. Mid-pandemic, mothers averaged 33 years old (*SD* = 5.31), and children averaged 7.87 years old (*SD* = 1.42). The analytic sample did not differ from the 175 dyads that initially participated (i.e., the T1 sample) on the above demographic variables, the location of their visit, or reports of their children’s internalizing and externalizing behaviors at T1 (*ps* > .05). Statistics for main study variables and potential covariates (i.e., pre-pandemic internalizing and externalizing behaviors [T1], mid-pandemic maternal depressive symptoms [T2], pre-pandemic child age [T1], days between visits, mid-pandemic cumulative trauma exposure [T2] variables are provided, stratified by sex, in Table 2. Independent sample *t*-tests revealed no significant differences by sex or by visit location in variables of interest [*ps* > .05]).

Associations between main study variables and potential covariates were explored and are reported in Table 3. Significant and positive relations were observed between pre- and mid- pandemic behaviors. Thus, we report on models controlling for pre-pandemic behaviors, consistent with previous research examining the moderating role of pre-disaster neurobiology on post-disaster outcomes (Mikolajewski & Scheeringa, 2018; Weissman et al., 2021). Of note, a similar pattern of results was observed for analysis without controlling for pre-pandemic behaviors.

Maternal depressive symptoms and cumulative trauma exposure were both positively and significantly associated with current internalizing and externalizing behaviors and were thus included in both models. As expected, baseline RSA was significantly and negatively associated with RSA reactivity and was thus included in reactivity models. Child age was included due to significant and positive associations with baseline RSA. Child sex was not covaried due to no significant differences by sex in variables of interest. Finally, days between visits was significantly and positively associated with COVID stress exposure, and thus covaried.

### Internalizing behaviors

At both pre- and mid-pandemic, *T* scores for internalizing behaviors predominately fell below borderline and clinical cutoffs. No main or interaction effects were observed between baseline RSA and COVID stress predicting to mid-pandemic internalizing behaviors, although there were significant main effects of pre-pandemic internalizing behaviors (95% C.I.s [.11–.46]) and cumulative trauma exposure (95% C.I.s [.06–.81]; see Table 4). Similarly, and contrary to hypotheses, no main effects of RSA reactivity or COVID stress were observed nor was an RSA reactivity by COVID stress interaction. There were significant main effects of pre- pandemic internalizing behaviors (95% C.I.s [.12–.47]) and cumulative trauma exposure (95% C.I.s [.03–.79]) predicting to children’s T2 internalizing behaviors (see Table 5).

### Externalizing symptoms

**Figure 3.**
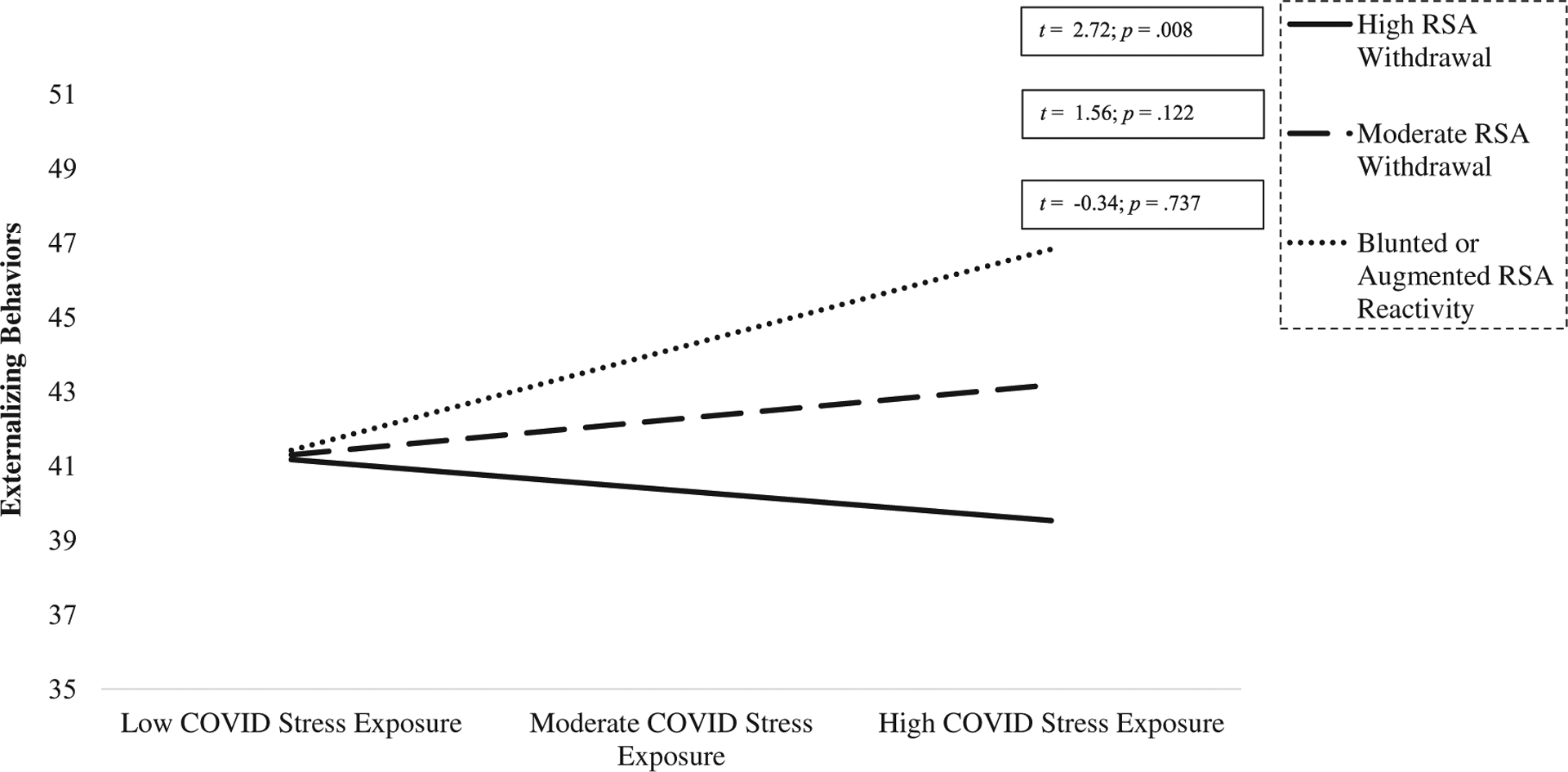
COVID stress exposure is positively associated with mid-pandemic externalizing behaviors for children exhibiting blunted or augmented RSA reactivity pre-pandemic. Values are graphed at −1 SD (−1.26), mean (−.60), and + 1 SD (.07; low) levels of RSA reactivity.

At both pre- and mid-pandemic, *T* scores for externalizing behaviors predominately fell below borderline and clinical cutoffs. No main effects or interaction effects were observed between baseline RSA and COVID stress; there were only significant main effects of pre- pandemic externalizing symptoms (95% C.I.s [.13–.43]) predicting to mid-pandemic externalizing symptoms (see Table 3). Regarding RSA reactivity, significant, positive main effects of COVID stress (95% C.I.s [.02–.86]) and child externalizing behaviors at pre- pandemic (95% C.I.s [.13–.42]) were observed for mid-pandemic child externalizing behaviors. This main effect was qualified by a significant COVID stress-by-RSA reactivity interaction predicting to mid-pandemic externalizing behaviors (95% C.I.s [.08–1.00]; *F* = 6.36; Table 5). For children exhibiting high RSA withdrawal (−1.26; +1SD) and moderate (−0.60; mean) RSA withdrawal, COVID stress was unrelated to externalizing behaviors. However, for children who exhibited blunted RSA reactivity or augmentation (.07; −1SD), consistent with hypotheses, COVID stress was significantly and positively associated with externalizing behaviors (see Fig. 3).

**Figure 4.**
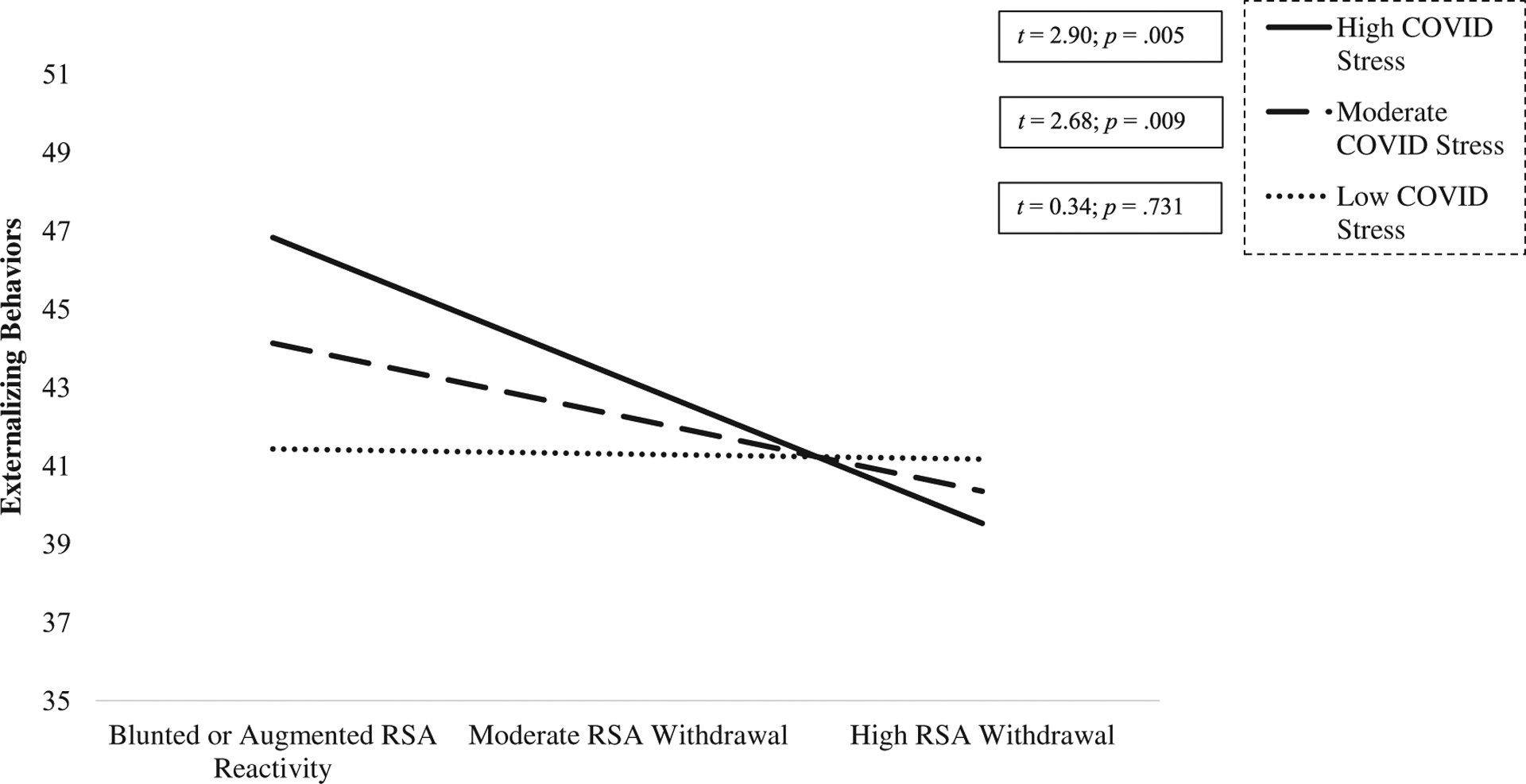
Children exhibiting pre-pandemic blunted or augmented RSA reactivity exposed to high or moderate levels of covid stress exhibited higher levels of externalizing behaviors. Values are graphed at −1 SD (3.75), mean (8.15), and + 1 SD (12.54) of COVID stress exposure.

To further decompose this interaction, we next examined conditional associations between RSA reactivity and externalizing behaviors at high (12.54; +1 S.D.), moderate (8.15; mean), and low (3.75; −1 S.D.) levels of COVID stress. For children exposed to low levels of COVID stress, RSA reactivity and externalizing behaviors were unrelated. However, for those exposed to high or moderate levels of COVID stress, higher levels of pre-pandemic RSA withdrawal buffered against the development of mid-pandemic externalizing behaviors, while blunted RSA reactivity or RSA augmentation exacerbated the risk for these behaviors (see Fig. 4).

## Discussion

The current study considered the co-contribution of COVID stress exposure and pre-pandemic physiological sensitivity on current internalizing and externalizing behaviors in a community-based sample of LIEM elementary school-aged children. Consistent with hypotheses, we found cross-sectional and positive associations between COVID stress and child externalizing behaviors. Importantly, we found that these associations were conditional, such that COVID stress and externalizing behaviors were related for children who exhibited low physiological sensitivity (i.e., blunted or augmented RSA reactivity) during a parent-child challenge in early childhood. Conversely, no associations were observed between COVID stress exposure and externalizing behaviors for children who exhibited high physiological sensitivity (i.e., RSA withdrawal in response to dyadic stress, suggesting physiological sensitivity in early childhood may act as a buffer. Our findings were also consistent with DS frameworks; associations between the moderator (RSA reactivity) and outcome (externalizing behaviors) were significant for children exposed to high and moderate, but not low, levels of COVID stress (Roisman et al., 2012). Taken together, these fully attenuated interactions provide evidence consistent with extant literature in similar age groups suggesting that low physiological sensitivity enhances risk for externalizing behaviors in contexts of stress (El-Sheikh et al., 2001; El-Sheikh & Hinnant, 2011; Katz, 2007; Salisbury et al., 2020).

Contrary to hypotheses and polyvagal, diathesis stress, and differential susceptibility theories, baseline RSA did not moderate associations between COVID-related stress exposure and mental health symptoms. This may be due to the community-based nature of our sample; a systematic review and meta-analysis of childhood adversity and RSA activity found that associations between baseline RSA and childhood adversity were stronger in clinical samples than in community samples (Wesarg et al., 2022). Contrary to hypotheses and DS or BSC frameworks, we also did not find associations between COVID stress exposure and internalizing behaviors at different levels of baseline RSA or RSA reactivity. Similarly, studies testing theories of neurobiological susceptibility in early and middle childhood found relations predicting to externalizing, but not internalizing behaviors (Obradović et al., 2010; Salisbury et al., 2020). Cross-sectional studies with adolescents, however, found partially attenuated interactions such that greater baseline RSA and RSA withdrawal increased vulnerability for internalizing behaviors in those exposed to high adversity (McLaughlin et al., 2014, 2015) and low supportive parenting (Mezulis et al., 2015). Finally, a similar longitudinal study testing neurobiological susceptibility models with adolescents found crossover interactions such that those with higher pre-pandemic PNS withdrawal exhibited the highest levels of emotional problems in contexts of high COVID stress, but the lowest levels of emotional problems in contexts of low COVID stress (Miller et al., 2021). Future research should continue exploring how the moderating role of RSA activity varies by developmental stage.

Although cumulative trauma exposure was significantly and positively associated with externalizing behaviors in bivariate correlations, in regression models including COVID stress as a predictor, it did not account for variance in externalizing behaviors. Contrary to hypotheses, we did not observe associations between COVID stress exposure and internalizing behaviors. Only pre-pandemic internalizing behaviors and cumulative trauma exposure significantly and positively predicted mid-pandemic internalizing behaviors. Similarly, in a longitudinal sample of LIEM mothers in New Orleans, exposure to and mental health during Hurricane Katrina predicted symptoms of psychological distress and posttraumatic stress during COVID (Zacher et al., 2023). Further, heightened pre-pandemic stress exposure was associated with both exposure to COVID stressors and to mental and physical health difficulties in Black Americans (Carter et al., 2021). In alignment with the COVID-19 family disruption model, results affirm that both proximal and distal exposures impact mental health outcomes, and their impact differs by symptom type. However, the present study was limited in its lumping methodology, which cannot isolate the effects of exposure to individual stressors both related and unrelated to COVID. Relatedly, future research with Black communities should measure stressors that disproportionately impact factors that interact with COVID stress exposures, such as those assessed in a supplemental module of the EPII assessing impact related to racial and ethnic discrimination (Yang et al., 2020). In sum, future research can build on the present study through utilizing dimensional analytic approaches (McLaughlin et al., 2021), measuring community-specific stressors, and using trauma screening measures that capture important features of the exposure such as frequency, chronicity, and severity of exposure (e.g., UCLA PTSD index; Kaplow et al., 2020).

### Clinical implications

Children with externalizing problems struggle with dysregulated anger and approach- related affect, which are often socialized and reinforced in relational contexts (Beauchaine, 2015). Blunted RSA activity during a dyadic stressor may reflect a child’s difficulty engaging, which could in turn prompt heightened engagement from the mother in attempts to draw the child in. In previous work, mother-child dyads where preschool-aged children had higher externalizing behaviors have shown discordance in mother-child RSA coregulation during similar dyadic tasks (Lunkenheimer et al., 2015; Lunkenheimer et al., 2018). To contextualize the adaptive value of RSA reactivity, future research should not only measure self-regulation within both members of the dyad, but also across them. For example, research examining parent-child coregulation during a challenging dyadic task in a sample of young children found that, in dyads with higher physiological synchrony, children exhibited RSA withdrawal while receiving critical feedback from their parents (Armstrong-Carter et al., 2021). Further, in a sample of preschool- aged children exhibiting disruptive behavior, RSA withdrawal in response to a dyadic challenge increased after participating in a parent-child intervention targeting children’s self-regulation and parent’s coercive parenting responses (*Incredible Years;* Bell et al., 2018). Taken together, this suggests that, for children at risk for externalizing behaviors, RSA withdrawal in response to a dyadic task may be indicative of self-regulatory capacities (e.g., engagement, active coping).

Alternatively, this could suggest that blunted RSA reactivity or RSA augmentation may be indicative of dysregulation, representing a child’s physiological disengagement during their parent’s coercive attempts at behavior modification. Future research can offer additional evidence to this theory through measuring child behavior to determine behavioral correlates of children’s RSA reactivity during a dyadic task.

The disparate outcomes we observed by level of stress exposure emphasizes the importance of research examining how systemic processes underly or exacerbate mental health difficulties and how changes at structural levels can buffer against these outcomes. Future research examining the role of physiological sensitivity in the etiology of stress-related mental health difficulties should also consider preventive interventions that decrease the level of environmental exposure. For example, previous research has found that policy solutions (e.g., food aid, stimulus checks) may partially alleviate symptom differences through reducing socioeconomic disparities in brain development (Weissman et al., 2023) and COVID-related stress exposure (Parenteau et al., 2022). The current study has implications for researchers, clinicians, and policy makers through identifying both individual and structural treatment targets to reduce stress-related suffering.

### Strengths, limitations, and future directions

The present study contains several limitations when considering generalizability of results and implications. First, the adaptive value of a physiological response is defined by how well-matched it is with a dyadic stress task. This stressor is well-validated in physiological research designed to evoke physiological reactivity that may generalize outside of laboratory settings and occur frequently in early childhood (e.g., receiving parental instruction when cleaning up toys or getting ready for school; Obradović et al., 2010). However, results may differ from studies where RSA was elicited using a different challenge task (e.g., cognitive vs interpersonal; Obradović et al., 2011) or emotionally evocative stimuli (Gatzke-Kopp et al., 2015). Second, we were unable to assess effects of sex due to power limitations, although doing so would potentially contribute to the extensive literature documenting such effects (El-Sheikh et al., 2001; Hinnant & El-Sheikh, 2013; Gray et al., 2017). Future research with adequate power should explore this important future direction.

Existing work measuring the cumulative effect of chronic stress exposure among Black Americans highlights how the risk for stress exposure is heightened in contexts of longstanding oppression (Carter et al., 2021). Relatedly, our sample was small and largely homogeneous regarding race and SES status, which limited our ability to disentangle SES status and race.

Future research that is adequately powered should do so, as these are often conflated in the literature due to a historical and contemporary policies rooted in racism and oppression placing disproportionate financial stress on Black Americans (e.g., housing segregation; Williams & Collins, 2001). Exposure to violence was also overrepresented in our sample, which may unfortunately generalize across similar samples – LIEM children face greater risk for exposure to several forms of violence (e.g., maltreatment, community violence, and interpersonal violence; Briggs-Gowan et al., 2010). Although our results may not generalize nationally, they offer important and timely contributions through investigating processes in early childhood which exacerbate risk for stress-related difficulties in a population of children that are both underrepresented in the research and face chronic and disparate exposure to stress.

Although several studies in the extant literature conceptualize fewer exposures as a positive environment (Gray et al., 2017; Miller et al., 2021; Obradović et al., 2010; Skowron et al., 2014), future research should include supportive influences to capture the full spectrum of environmental experience. Another longitudinal study found that baseline RSA collected in infancy was negatively associated with aggressive behaviors in early childhood for children in supportive contexts, but there was no relation in unsupportive environments (Eisenberg et al., 2012). Relatedly, future research should integrate supportive social and environmental factors (e.g., social support, religiosity and spirituality, ethnic and racial socialization, and family values and rituals), which have buffered against the development of psychopathology following stress exposure in other samples of predominantly Black children (Stern et al., 2021; Tyrell & Masten, 2022).

## Conclusion

We found that blunted or augmented RSA reactivity during a parent-child challenge in early childhood, a rapid period of ANS development, has a cascading influence impacting the development of externalizing behaviors, and that this influence is most consequential in contexts of high stress. Findings have critical implications both for the links between COVID stress exposure and child mental health and for the moderating role of physiological self-regulation in early childhood in these relations. Findings highlight the important, prospective role of RSA activity both in early childhood and within the caregiving context in preventing post-exposure outcomes. Further, evidence supporting the idea that children’s risk for externalizing behaviors is increased when exposed to moderate and high levels of COVID stress highlights the need to disseminate preventive interventions in and target resources toward communities facing disparate stress exposure. Despite the limitations previously noted, the present study advances developmental science, extending the DS model to child mental health during the COVID-19 pandemic.

## Figures and Tables

**Table 1.** Sociodemographic characteristics of mothers and children

	Children		Mothers	
Characteristic	*n*	%	*n*	%
Sex				
Female	40	49%	81	100
Male	41	51%	0	0
Race				
Asian	2	2.20%	2	2.20%
Black / African American	65	79.80%	63	78.70%
White	7	9.00%	12	14.60%
Other	7	9.00%	4	4.50%
Ethnicity				
Hispanic or Latino/a	13	15.50%	8	9.10%
Not Hispanic or Latino/a	68	84.50%	73	90.90%

*Note. N* = 81 mother-child dyads. Pre-pandemic (Time 1; T1), children were on average 4.28 (*1.30)* years old and mothers were on average 30.08(*5.31)* years old. Mid-pandemic (Time 2; T2), children were on average 7.87(*1.42)* years old and mothers were on average 33.02 (*4.28)* years old.

**Table 2.** Results of T − tests exploring differences in study variables between girls and boys

		Girls	Boys			
Variable	Range	Mean (*SD*)	Mean (*SD*)	*t*(81)	*p*	Cohen’s *d*
COVID Stress (T2)	0 – 21	7.47(4.63)	8.74(4.30)	1.36	.18	.29
Baseline RSA (T1)	1.89 – 9.26	6.16(1.54)	6.35(1.63)	.54	.60	.12
RSAA (T1)	− 2.01 −1.25	− 0.53(.65)	− 0.64(0.68)	− .73	.47	.16
Internalizing (T2)	29 – 67	39.41(9.24)	38.85(8.67)	.18	.86	.04
Externalizing (T2)	30 – 67	40.91(9.37)	45.09(8.90)	.44	.66	.09
Internalizing (T1)	29 – 81	46.98(11.02)	47.65(10.92)	− .03	.98	.01
Externalizing (T1)	28 – 83	45.13(11.45)	46.25(11.29)	.18	.86	.04
Maternal Depression (T2)	1 – 69	18.63(17.50)	17.00(17.27)	− .44	.66	.09
Cumulative Trauma (T2)	0 – 13	2.69(3.15)	2.41(3.23)	− .41	.68	.09
Child Age (T1)	36 – 70	50.14(9.33)	51.11(9.62)	.48	.63	.10
Days Between Visits	752 – 1,859	1,336.63(292.80)	1,300.87(301.86)	− .57	.57	− .12

*Note. N* = 81 children, *n* = 40 girls; 49% and *n* = 41 boys; 51%. *T1* = Time 1, *T2* = Time 2, *RSAΔ* = Difference in RSA from baseline to task.

**Table 3.** Correlations among study variables and covariates

Variable (*n* = 81)	M(*SD*)	1	2	3	4	5	6	7	8	9	11
1. COVID Stress (T2)	8.16(4.47)	–									
2. RSAΔ (T1)	6.26(1.58)	.11	–								
3. Externalizing (T2)	5.66(6.04)	**.38** **	**.22** *	–							
4. Externalizing (T1)	10.29(8.69)	**.37** **	.08	**.50** **	–						
5. Internalizing (T2)	4.03(5.03)	**.34** **	.07	**.60** **	**.41** **	–					
6. Internalizing (T1)	7.38(5.78)	.24*	− 0.05	**.30** **	**.76** **	**.44** **	–				
7. Maternal Depression (T2)	17.66(17.24)	**.36** **	.01	**.24** *	**.28** **	**.31** **	.11	–			
8. Cumulative Trauma (T2)	2.60(3.13)	**.46** **	.00	**.27** *	**.36** **	**.41** **	**.23** *	**.34** **	–		
9. RSA Baseline (T1)	6.26(1.58)	.00	**− .25** **	.08	.03	.11	.05	.05	.07	–	
11. T1 Child Age (months)	50.64(9.44)	− .08	− .02	− .12	**− .16** *	− .13	.03	− .02	− .04	**.22** **	–
12. Days Between Visits	1318(296.44)	**− .27** **	.04	− .08	− .05	.07	.07	− .03	− .02	− .08	.16

*Note*. T1 = Pre−pandemic (Time 1), T2 = Mid-pandemic (Time 2), RSAΔ = Difference in RSA from baseline to task.

* *p* < .05;

** *p* < .01.

**Table 4.** Standardized regression coefficients for moderator analysis: baseline RSA and COVID stress on behavior problems at time 2

		Externalizing				Internalizing				
				95% CI					95% CI	
Baseline RSA	β	*t*(81)	*p*	*LL*	*UL*	β	*t*(81)	*p*	*LL*	*UL*
Child Age	− .05	− 1.19	.24	− .13	.03	− .06	− 1.81	.11	− .13	.01
Behavior Problems (T1)	.28	3.75	< .001*	.13	.43	.29	3.29	**.002** **	.11	.46
Maternal Depression (T2)	.03	− 1.19	.24	− .05	.11	.04	1.27	.21	− .02	.11
Days Between Visits	.00	.48	.63	− .00	.00	.00	1.28	.21	− .00	.01
Cumulative Trauma (T2)	.00	.00	1.00	− .45	.45	.44	2.33	.02*	.06	.81
RSAb (T1)	1.29	1.49	.14	− .44	3.02	.86	1.18	.24	− .59	2.31
COVID Stress (T2)	.85	1.44	.15	− .33	2.03	.42	.86	.39	− .56	1.40
RSAb × COVID Stress	− .11	.48	.63	− .29	.07	− .06	− .75	.45	− .21	.09
*ΔR* ^*2*^			.01					.00		
Total *R*^*2*^			.33					.36		

*Note. T1* = Time 1; *T2* = Time 2; *RSAb* = Baseline RSA; *CI* = Confidence Interval; *LL* = Lower limit; *UL* = Upper limit.

*p* < .05;

** *p* < .01;

*** *p* < .001.

**Table 5.** Standardized regression coefficients for moderator analysis: RSA reactivity and COVID stress exposure on behavior problems

		Externalizing				Internalizing				
				95% CI					95% CI	
RSA Reactivity	β	*t*(81)	*p*	*LL*	*UL*	β	*t*(81)	*p*	*LL*	*UL*
Child Age	− .04	− .90	.37	− .12	.04	− .06	− 1.64	.11	− .12	.01
Behavior Problems (T1)	.28	3.84	**.003** **	.13	.42	.29	3.39	**.001** **	.12	.47
Maternal Depression (T2)	.06	1.41	.16	− .02	.13	.05	1.44	.15	− .02	.12
Days Between Visits	.00	.19	.85	− .00	.00	.00	1.10	.27	− .00	.00
Cumulative Trauma (T2)	− .07	− .33	.74	− .52	.37	.41	2.16	**.03** *	.03	.79
RSAb (T1)	.57	1.56	.12	− .16	1.31	.45	1.40	.16	− .19	1.09
*Δ*RSA (T1)	− 1.92	− 1.10	.28	− 5.40	1.56	− .68	− .45	.66	− 3.69	2.34
COVID Stress (T2)	.44	2.10	**.04** *	.02	.86	.16	.88	.38	− .20	.52
RSAΔ × COVID Stress	.54	2.32	**.02** *	.08	1.00	.20	1.01	.32	− .20	.60
*ΔR* ^*2*^			**.05** *					.01		
Total *R*^*2*^			.39					.38		

*Note. T1* = Time 1; *T2* = Time 2; *RSAb* = Baseline RSA; *RSAΔ* = Difference in RSA from baseline to task; *CI* = Confidence Interval; *LL* = Lower limit; *UL* = Upper limit.

* *p* < .05;

** *p* < .01;

*** *p* < .001.
